# Purinergic Signaling in Swallowing Reflex Initiation: Mechanisms and Therapeutic Implications for Oropharyngeal Dysphagia—A Narrative Review

**DOI:** 10.3390/cells14221795

**Published:** 2025-11-14

**Authors:** Junrong Qi, Mohammad Zakir Hossain, Hiroshi Ando, Rita Rani Roy, Junichi Kitagawa

**Affiliations:** 1 Department of Orthodontic Clinic, Matsumoto Dental University Hospital, 1780 Gobara Hirooka, Shiojiri 399-0781, Nagano, Japan; kunyou.ki@mdu.ac.jp; 2 Department of Oral Physiology, School of Dentistry, Matsumoto Dental University, 1780 Gobara Hirooka, Shiojiri 399-0781, Nagano, Japan; rani.roy.rita@mdu.ac.jp; 3 Department of Biology, School of Dentistry, Matsumoto Dental University, 1780 Gobara Hirooka, Shiojiri 399-0781, Nagano, Japan; hiroshi.ando@mdu.ac.jp

**Keywords:** swallowing reflex, purinergic signaling, adenosine triphosphate (ATP), P2X3 receptor, P2Y1 receptor, oropharyngeal dysphagia

## Abstract

The swallowing reflex is a highly coordinated process that is essential for safe bolus transit and airway protection. Although its neurophysiological framework has been extensively studied, the molecular mechanisms underlying reflex initiation remain incompletely understood, limiting targeted therapies for oropharyngeal dysphagia. Recent evidence implicates purinergic signaling as a key mediator of swallowing initiation, particularly through ATP release from taste buds and neuroendocrine cells in the hypopharyngeal and laryngeal mucosa. Experimental studies in mice demonstrate that water, acidic, and bitter chemical stimuli induce ATP release, activating purinergic receptors (P2X2, P2X3, heteromeric P2X2/P2X3, and P2Y1) on afferent sensory fibers. This receptor activation enhances input to the brainstem swallowing central pattern generator, initiating reflexive swallowing. Genetic ablation of purinergic receptor-expressing neurons or epithelial sentinel cells, as well as pharmacological antagonism of P2X or P2X3 receptors, markedly attenuates these responses. Furthermore, exogenous ATP or selective P2X3 agonists applied to swallowing-related mucosa evoke swallowing reflexes in an animal model, underscoring translational potential. While the precise upstream receptor mechanisms for water- and acid-induced ATP release, as well as species-specific differences, remain to be clarified, targeting purinergic pathways may represent a novel physiologically grounded therapeutic strategy for restoring swallowing function in patients with oropharyngeal dysphagia.

## 1. Introduction

The swallowing reflex is a vital and highly coordinated physiological process that facilitates the safe and efficient transport of food, liquids, and saliva from the oral cavity to the esophagus and, ultimately, to the stomach [1,2,3,4,5]. This reflex comprises complex neural and muscular events involving oral, pharyngeal, and esophageal phases. While under voluntary control in its initial stage, the reflex transitions into an involuntary, centrally mediated response that is critical for the seamless passage of ingested sub-stances [1,2,3,4,5]. In addition to its fundamental role in digestion, the swallowing reflex serves a crucial protective function by preventing the inadvertent entry of food, liquid, or saliva into the airway, thereby reducing the risk of aspiration and subsequent respiratory complications such as aspiration pneumonia [1,2,3,4,5,6].

The reflex can be elicited by various stimuli—including water [7,8,9], mechanical stimuli (e.g., touch or air puffs) [10,11,12,13,14], chemical stimuli (e.g., citric acid) [7,15,16,17], and electrical stimuli [10,18,19,20,21,22]—applied to the mucosal surfaces of the oropharyngeal and laryngeal regions. Sensory information generated at the level of the mucosa is transmitted via afferent fibers of cranial nerves such as the glossopharyngeal and vagus nerve to the central pattern generator for swallowing (sCPG) in the brainstem, as well as to cortical and subcortical swallowing-related regions [1,2,3,4,5].

Impairments or delays in the initiation or execution of the swallowing reflex—commonly referred to as oropharyngeal dysphagia—can be caused by various factors, including aging, neurological and neurodegenerative diseases (e.g., Parkinson’s disease, Alzheimer’s disease, amyotrophic lateral sclerosis), neuromuscular disorders (e.g., myasthenia gravis), and cerebrovascular accidents (e.g., stroke) [23,24,25,26,27,28,29,30]. Dysphagia not only compromises nutritional intake but also significantly increases the risk of aspiration, dehydration, and malnutrition, thereby reducing quality of life and increasing morbidity and mortality [31,32,33,34]. Despite its clinical importance, the molecular mechanisms underlying initiation of the swallowing reflex remain poorly defined, hindering the development of targeted therapeutic interventions [5,35]

In recent years, purinergic signaling has emerged as a promising area of investigation in the context of sensory transduction [36,37,38,39]. This signaling system, mediated by extracellular purine nucleotides and nucleosides—particularly adenosine triphosphate (ATP)—extends beyond its classical role as an intracellular energy molecule to function as a key extracellular messenger [36,37,38,39]. Under physiological and pathophysiological conditions, ATP can be released from various cell types, including epithelial cells, sensory neurons, and glial cells, into the extracellular milieu in response to mechanical stress, chemical stimulation, or cellular damage. Once released, ATP binds to purinergic receptors expressed on nearby or distant cells, initiating downstream signaling cascades that influence a variety of biological responses [36,37,38,39]. Purinergic receptors are broadly classified into two families: P1 receptors, which are G protein-coupled receptors activated by adenosine, and P2 receptors, which respond to ATP and other nucleotides [37,38,40]. The P2 receptor family is further subdivided into ionotropic P2X receptors—ligand-gated ion channels that mediate rapid cation influx (e.g., Na^+^ and Ca^2+^)—and metabotropic P2Y receptors, which are also G protein-coupled receptors and signal through second messenger systems such as inositol triphosphate or cyclic adenosine monophosphate. In mammals, seven P2X receptor subtypes (P2X1–P2X7) and eight P2Y receptor subtypes (P2Y1, P2Y2, P2Y4, P2Y6, and P2Y11–P2Y14) have been identified [37,38,40].

Recent experimental findings suggest that ATP released in response to water, acidic, or bitter stimulation of the pharyngeal and laryngeal mucosa activates purinergic receptors, particularly those expressed on afferent sensory nerves [41,42,43]. This activation may enhance afferent signaling to the brainstem swallowing centers and cortical and subcortical swallowing-related networks, thus facilitating the initiation of the swallowing reflex. A pharmacological study using purinergic receptor agonists and antagonists reported modulation of the swallowing reflex [44]. Moreover, immunohistochemical studies have identified the expression of purinergic receptors in peripheral sensory structures implicated in swallowing control [44,45,46,47,48].

This narrative review aims to summarize recent advances in understanding the role of purinergic signaling in the initiation of the swallowing reflex. A comprehensive literature search was conducted to identify relevant studies, using PubMed, Embase, Web of Science, ScienceDirect, and Google Scholar. Search terms were used in various combinations and included purinergic signaling, ATP, ATP receptors, P2X receptors, P2Y receptors, taste cells and swallowing, swallowing reflex, superior laryngeal nerve, oropharyngeal dysphagia, and therapeutics for dysphagia. Additional references were identified through manual searches of key journals, citation tracking, and review of reference lists from relevant articles. Only studies published in English were considered, and both preclinical and clinical studies were included to provide a comprehensive overview of mechanisms and potential therapeutic strategies. The review was prepared in accordance with the SANRA (Scale for the Assessment of Narrative Review Articles) guidelines [49] to ensure clarity, methodological transparency, and scientific rigor.

## 2. Purinergic Receptors in Peripheral Swallowing-Related Regions

Previous studies have reported the presence of purinergic receptors in peripheral swallowing-related regions [41,43,44,45,46,47,48,50,51] (Table 1). Expression of P2X3 and P2X2 receptors has been observed in intraepithelial and subepithelial nerve fibers, as well as in fibers associated with chemosensory and neuroendocrine cells within the laryngeal and pharyngeal regions of rats and mice [41,44,45,46,47,48]. These receptors are also found on nerve fibers associated with taste buds across multiple species, including rats, mice, monkeys, and humans [43,44,45,50,51,52,53,54]. Additionally, a study identified P2Y1-expressing nerve fibers that innervate the laryngeal taste buds and surrounding epithelium in mice [41].

## 3. Involvement of Purinergic Signaling in Water and Acid-Induced Swallowing Reflex

Recent evidence has highlighted a novel role for purinergic signaling, particularly through taste buds and P2Y1-receptor-expressing neurons, in mediating the swallowing reflex induced by water and acid in mice [41]. A comprehensive study employing single-cell RNA sequencing and clustering analysis identified approximately 37 distinct classes of vagal and glossopharyngeal sensory neurons within the nodose-petrosal-jugular ganglionic complex (NPJc) [41]. Using Cre-driver lines crossed with a Cre-dependent channelrhodopsin allele (*loxP-ChR2*), the researchers performed optogenetic stimulation targeting small subsets of these neuronal populations. Among these, optogenetic activation of P2Y1-expressing neurons robustly evoked swallowing reflexes. Targeted ablation of vagal P2Y1 neurons selectively attenuated water- and citric acid-induced swallowing reflexes, while leaving mechanical and high-salt-induced reflexes unaffected. To map the anatomical distribution of P2Y1-expressing fibers, Cre-dependent adeno-associated viruses were injected into the NPJc of *P2Y1-ires-Cre* mice. This revealed dense and intricate P2Y1-labeled projections in the ciliated epithelium of the laryngeal surface, including the epiglottis and subglottic regions. Additionally, distinctive P2Y1-positive nerve endings were observed in the squamous epithelium near the vocal folds, arytenoid cartilages, and aryepiglottic folds [41]. These fibers often formed corpuscle-like structures that were found in close apposition to laryngeal taste buds, as confirmed by immunohistochemical labeling for the taste cell marker keratin 8.

The innervation of laryngeal epithelial regions, including taste buds, by P2Y1-expressing fibers suggests the potential involvement of upstream sentinel epithelial cells that detect water and/or acid stimuli and subsequently transmit signals to P2Y1 sensory neurons. To test this hypothesis, the study [41] utilized *Krt8-Cre^ER^; loxP-ChR2* mice, in which channelrhodopsin-2 was broadly expressed in the ciliated epithelium and selectively in taste buds within the squamous epithelium, but not in vagal sensory neurons. Optogenetic stimulation of the inferior edge of the arytenoids and vocal folds—though not the upper trachea, posterior oral cavity, or NPJc—elicited swallowing responses in these mice. This finding supports the idea that epithelial stimulation in the larynx is sufficient to initiate the neural circuitry underlying the swallowing reflex, with P2Y1 neurons acting downstream of the epithelial cells.

**Figure 1:**
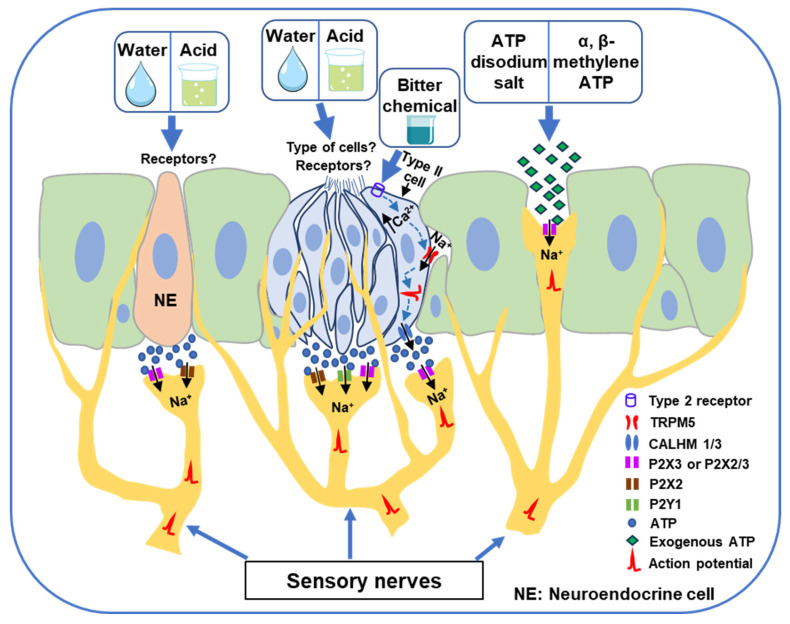
Schematic representation of the involvement of purinergic signaling in triggering the swallowing reflex based on published studies [41,42,43,44]. Application of water or acidic solutions to peripheral swallowing-related regions activates hypopharyngeal and laryngeal taste bud cells (specific cell types and receptors remain unidentified) and neuroendocrine cells (receptors remain unidentified), leading to ATP release. ATP stimulates purinergic receptors (P2X2, P2X3, heteromeric P2X2/3, and P2Y1) on sensory nerves, inducing cation influx (e.g., Na^+^), action potential generation, and enhanced sensory input. Bitter compounds activate type 2 taste receptors (T2Rs) on type 2 taste bud cells, increasing intracellular Ca^2+^, which activates transient receptor potential melastatin 5 (TRPM5) channels, promotes cation influx, and triggers ATP release via calcium homeostasis modulator 1/3 (CALHM1/3) channels. Direct application of exogenous ATP (e.g., ATP disodium salt) similarly activates purinergic receptors on sensory nerves, leading to their excitation. These sensory inputs are transmitted to sCPG and higher brain centers, facilitating initiation of the swallowing reflex.

To further examine the involvement of ATP signaling, the study [41] assessed swallowing reflexes in *P2X2/P2X3* double-knockout mice. In these animals, water failed to induce a swallowing reflex, and the response to acid was significantly diminished, whereas reflexes triggered by mechanical or high-salt stimuli remained unchanged. These results underscore the critical role of ATP-mediated purinergic signaling in water- and acid-induced swallowing reflexes. Collectively, the findings suggest that water and acidic stimuli activate laryngeal taste bud cells, leading to ATP release, which then stimulates P2X2, P2X3 and P2Y1-expressing afferent neurons that transmit signals to the sCPG responsible for initiating the swallowing reflex (Figure 1). However, the specific receptors and taste bud cell types activated by water and acid stimuli remain to be identified.

Another elegant study demonstrated the involvement of neuroendocrine (NE) cells—specialized epithelial cells—in mediating water- and acid-induced swallowing reflexes via purinergic signaling [42]. The study utilized *Ascl1^CreERT2^; R26^LSL-tdTomato^* mice, in which cells expressing the transcription factor achaete-scute family basic helix-loop-helix transcription factor 1 (*Ascl1*, a lineage-defining transcription factor for NE cells [55]) can be labeled following tamoxifen-induced Cre recombination, resulting in tdTomato, a fluorescent protein, being expressed in these cells and their progeny. This model enabled specific labeling of NE cells within the airway. Using this mouse model, the researchers labeled and isolated NE cells in the trachea and larynx. Calcium imaging of tissue slices revealed that both water and acid robustly activated NE cells in these regions. In dissociated NE cells, exposure to acidic stimuli (pH < 4) induced substantial calcium influx. Additionally, tracheal NE cells exhibited intrinsic sensitivity to water, responding to hypo-osmotic stimuli below 75 mOsm. Exposure to a water stimulus triggered ATP release from tracheal and laryngeal NE cells.

Optogenetic stimulation of NE cells in *Ascl1^CreERT2^* mice crossed with *Rosa^26LSL-ReaChr^* or *Rosa^26LSL-ChR2^* led to increased activity in the superior laryngeal (SLN) and recurrent laryngeal nerves which innervate the larynx and trachea [42]. When a P2X receptor antagonist (pyridoxal-phosphate-6-azophenyl-2′,4′-disulfonic acid) was applied during optogenetic stimulation, SLN and recurrent laryngeal activity were significantly reduced, indicating that ATP release from NE cells activates afferent nerve fibers through purinergic mechanisms.

Furthermore, optogenetic activation of NE cells in the larynx, upper trachea, and mid-trachea in *Ascl1^CreERT2^*; *Rosa26^LSL-ChR2^* mice reliably evoked swallowing reflexes [42]. Genetic ablation of NE cells using *Ascl1^CreERT2^*; *Rosa26^lsl-Diphtheria Toxin A^* (*lsl-DTA*) and *Ascl1^CreERT2^*; *Rosa26^lsl-Diphtheria Toxin A Receptor^* (*lsl-DTR*) mice significantly diminished water- and acid-induced swallowing responses. These findings provide compelling evidence that water and acidic stimuli activate NE cells in the larynx and trachea, leading to ATP release, which then activates ATP receptors (P2X receptors) on afferent sensory fibers to ultimately trigger the swallowing reflex (Figure 1). Nevertheless, the exact receptors activated by water or acid stimuli remain unidentified.

## 4. Involvement of Purinergic Signaling in Bitter Chemical Substance-Induced Swallowing Reflex

A recent study demonstrated that hypopharyngeal type II taste cells—a purinergic subclass of Pou2f3^+^ chemosensory cells—are involved in triggering the swallowing reflex in response to bitter chemical substances [43]. Application of bitter-tasting chemical agonists for type 2 taste receptors —specifically cycloheximide and denatonium—to the lumen of the hypopharynx elicited the swallowing reflex. This response was significantly attenuated by AF-353, a selective antagonist of P2X3 and P2X2/3 receptors, indicating a key role for purinergic signaling in this process.

Optogenetic activation of hypopharyngeal and laryngeal Pou2f3^+^ chemosensory cells evoked responses in the SLN in mice expressing ChR2 under the control of either the *Calhm1* or *Pou2f3* promoter (*Calhm1-ChR2* or *Pou2f3-ChR2*) [43]. This optically evoked SLN activity was suppressed by AF-353, further supporting the involvement of purinergic receptors. Likewise, topical application of bitter chemical substances increased SLN activity, which was abolished in *Calhm3* knockout (*Calhm3*^Tom/Tom^) mice. These findings indicate that purinergic signaling mediates communication between Pou2f3^+^ chemosensory cells and SLN-afferent neurons.

Optogenetic stimulation of Pou2f3^+^ chemosensory cells in *Calhm1-ChR2* and *Pou2f3-ChR2* mice also induced the swallowing reflex, which was reversibly inhibited by topical AF-353 [43]. Notably, the swallowing reflex induced by bitter chemical substances was absent in *Pou2f3* knockout mice, demonstrating the essential role of these cells. Moreover, knockout of *Calhm3* abolished the bitter substance-induced swallowing reflex, underscoring the importance of channel synapse-mediated ATP release in this process. The study [43] further revealed that deletion of TRPM5 also abolished the reflex. Collectively, these findings suggest that activation of type 2 taste receptors and downstream signaling involving TRPM5 and channel synapses in hypopharyngeal type II taste cells leads to ATP release, which activates P2X3 or P2X2/3 receptors on SLN-afferent fibers (Figure 1). This purinergic excitation of sensory neurons activates the sCPG in the brainstem, ultimately triggering the swallowing reflex. The sCPG is a neural network responsible for generating the basic swallowing pattern [3,4]. It comprises dorsal and ventral neuronal groups. The dorsal group, located within the nucleus of the solitary tract (NTS) and adjacent reticular formations, is involved in generating, shaping, and timing sequential or rhythmic swallowing [3,4]. The ventral group, situated adjacent to the nucleus ambiguous, distributes the swallowing drive to the motor neurons of several cranial nerves, including the trigeminal, facial, glossopharyngeal, vagus, and hypoglossal nerves [3,4]. In this context, previous studies have reported the expression of P2X receptors in the NTS [56,57] and activation of these receptors modifies the electrical activity of NTS neurons, primarily by influencing glutamate release from presynaptic terminals [58,59,60]. Glutamate is a major excitatory neurotransmitter that triggers the swallowing reflex [3,4,61]. Therefore, excitation of presynaptic P2X receptors located on the nerve terminals of sensory neurons in the NTS can activate the sCPG to initiate the swallowing reflex.

## 5. Exogenous ATP Application to the Swallowing-Related Regions Triggers Swallowing Reflexes

A recent study reported that topical application of exogenous ATP (ATP disodium salt) to peripheral swallowing-related regions innervated by the SLN induces the swallowing reflex in rats [44]. Exogenous ATP application facilitated reflex initiation in a dose-dependent manner, indicating its excitatory effect on sensory pathways involved in swallowing.

To clarify the receptor mechanisms underlying exogenous ATP-induced reflexes, the study [44] investigated the role of P2X3 receptors. Immunohistochemical analysis revealed that P2X3 receptors were predominantly localized to nerve fibers within SLN-innervated swallowing-related tissues, including both intraepithelial and subepithelial nerves, as well as nerve fibers associated with taste-bud-like structures. Additionally, retrograde tracing of SLN-afferent neurons showed that approximately 40% expressed P2X3, with most of these being medium- to small-diameter neurons, consistent with a sensory neuronal phenotype.

Importantly, topical pretreatment with a P2X3 receptor antagonist (gefapixant) significantly attenuated ATP-induced swallowing reflexes, confirming the involvement of P2X3 in mediating the response [44]. Furthermore, application of a P2X3 receptor agonist (α, β-methylene ATP)—more specific than ATP—also facilitated swallowing reflexes in a dose-dependent manner, reinforcing the role of P2X3 activation in triggering the reflex. These findings strongly suggest that exogenous ATP activates P2X3 receptors on SLN-afferent fibers in peripheral swallowing-related regions to initiate the swallowing reflex (Figure 1). The study also raises the possibility that exogenous ATP or P2X3 receptor agonists could be explored as potential therapeutic agents for the management of oropharyngeal dysphagia.

## 6. Clinical Implications of Purinergic Signaling in Triggering the Swallowing Reflex

Current clinical management of oropharyngeal dysphagia primarily relies on compensatory strategies, such as modification of food viscosity, texture, or volume, and swallowing maneuvers including postural adjustments (e.g., chin tuck or head tilt) and targeted exercises [26,62,63,64,65,66]. Although these interventions aim to mitigate the functional impact of dysphagia, their efficacy in restoring impaired swallowing physiology remains limited [26,62,63,64,65,66,67,68]. As such, there is an urgent need to develop novel therapeutic approaches grounded in the underlying neurophysiological mechanisms of swallowing. In recent studies, chemical neurostimulation targeting peripheral chemosensory ion channels—such as transient receptor potential (TRP) channels—has been reported to show promising outcomes in both preclinical and clinical settings [5,11,69,70,71,72,73,74,75,76,77,78,79,80,81]. These approaches have demonstrated improvements in swallowing safety, efficiency, and neurophysiological function among individuals with oropharyngeal dysphagia [5,35,74,75,76]. In this context, emerging evidence supporting the role of purinergic signaling in triggering the swallowing reflex introduces an additional therapeutic target. The demonstrated ability of exogenous ATP and P2 receptor agonists (e.g., P2X3 agonist) to enhance swallowing reflexes suggests their potential as chemical neurostimulants to augment swallowing function [44]. Such pharmacological interventions may offer a novel and physiologically grounded strategy for improving dysphagia management, and warrant further investigation in clinical trials. Moreover, dysphagia is a major complication in neurodegenerative and neuroinflammatory diseases, such as amyotrophic lateral sclerosis and Alzheimer’s disease, in which dysarthria (difficulty in speech production) often coexists [82,83,84,85]. Recent findings indicate that altered purinergic signaling contributes to the pathogenesis of these disorders [86,87,88,89,90,91,92,93]. Therefore, targeting purinergic pathways may offer a therapeutic benefit through modulation of neurosensory and neuromotor mechanisms underlying these symptoms. Further studies are warranted to clarify the translational potential of these findings and to establish their clinical applicability.

## 7. Perspectives

Although substantial evidence indicates that water and acid stimulate taste buds [41] and neuroendocrine cells [42] in peripheral swallowing-related regions, the specific receptors activated on these cells to mediate the responses remain unidentified [94,95,96]. It also remains to be clarified whether water and acid activate the same type of taste receptor cells in the laryngeal taste buds, as has been suggested for lingual taste buds [97], or whether acid directly activates neurons through pH-sensitive channels (e.g., TRPV1 or acid-sensing ion channels), as proposed in a previous study [98]. Additionally, the mechanisms underlying swallowing reflexes induced by high salt concentrations and mechanical stimuli remain to be elucidated [94]. Furthermore, while the involvement of purinergic signaling in water-, acid-, and bitter chemical-induced swallowing reflexes has been demonstrated in mice, further investigations are needed to determine whether similar mechanisms operate in other species. Species-specific differences in triggering swallowing reflexes are evident; for instance, the TRPV1 channel activator capsaicin and the TRPA1 channel activator allyl isothiocyanate fail to induce the swallowing reflex in mice [41] but elicit robust responses in rats [11,78,99,100]. Similarly, in humans, TRP channel activators have been shown to facilitate swallowing [35,71,80,101,102,103]. These observations underscore the need to determine whether other species rely predominantly on purinergic signaling, alternative signaling pathways, or an integrated combination of mechanisms to mediate swallowing reflexes induced by water, acid, and bitter stimuli.

## 8. Conclusions

Emerging evidence highlights purinergic signaling as a pivotal mechanism in initiating the swallowing reflex, with ATP released from taste bud and neuroendocrine cells activating P2X2, P2X3, heteromeric P2X2/P2X3, and P2Y1 receptors on afferent sensory fibers (Figure 1). This receptor activation enhances sensory input to the brainstem swallowing central pattern generator and higher brain centers, facilitating reflexive responses to water, acid, and bitter stimuli. Moreover, exogenous application of ATP or selective P2X3 agonists has been shown to evoke swallowing reflexes, underscoring the translational potential of targeting purinergic pathways. These findings not only advance understanding of fundamental sensory transduction mechanisms in swallowing-related tissues but also provide a promising foundation for novel therapeutic strategies. In particular, pharmacological agents that modulate purinergic signaling may serve as effective chemical neurostimulants to restore or enhance swallowing function in individuals with oropharyngeal dysphagia. Further clinical studies are warranted to evaluate the efficacy, safety, and applicability of such interventions in therapeutic settings.

## Figures and Tables

**Table 1:** Presence of purinergic receptors in peripheral swallowing-related regions.

Purinergic Receptors	Regions	Localization	Species	Ref.
**P2X3**	Larynx (epiglottis, arytenoid, glottic and subglottic regions)	Intraepithelial ramified nerve fibers Nerve fibers associated with chemosensory cells and neuroendocrine cells Nerve fibers associated with taste buds	Rats	[45]
	Larynx	Nerve fibers associated with taste buds	Mice	[50]
	Larynx (epiglottis)	Intraepithelial nerve fibers	Rats	[46]
	Trachea	Intraepithelial and subepithelial nerve fibers	Rats	[47]
	Pharynx	Intraepithelial nerve fibers	Rats	[48]
	Laryngopharynx and associated laryngeal regions	Intraepithelial and subepithelial nerve fibers Nerve fibers associated with taste buds	Rats	[44]
	Hypopharynx	Nerve fibers contacting Pou2f3^+^ (POU class 2 homeobox factor 3) chemosensory cells	Mice	[43]
	Larynx	Nerve fibers contacting Pou2f3^+^ chemosensory cells	Mice	[43]
	Larynx	Nerve fibers associated with taste buds	Humans	[51]
	Larynx	Nerve fibers associated with taste buds	Monkeys	[51]
	Larynx	Nerve fibers associated with taste buds	Mice	[51]
	Back of the tongue	Nerve fibers associated with circumvallate taste buds	Rats	[52,53]
**P2X2**	Larynx (epiglottis, arytenoid region, glottic and subglottic regions)	Intraepithelial ramified nerve fibers Nerve fibers associated with chemosensory cells and neuroendocrine cells Nerve fibers associated with taste buds	Rats	[45]
	Larynx	Nerve fibers associated with taste buds	Mice	[50]
	Trachea	Intraepithelial and subepithelial nerve fibers	Rats	[47]
	Hypopharynx	Nerve fibers contacting Pou2f3^+^ chemosensory cells	Mice	[43]
	Larynx	Nerve fibers contacting Pou2f3^+^ chemosensory cells	Mice	[43]
	Larynx	Nerve fibers associated with taste buds	Mice	[51]
	Back of the tongue	Nerve fibers associated with circumvallate taste buds	Rats	[53,54]
**P2Y1**	Larynx	Nerve fibers innervating the epithelium Nerve fibers associated with taste buds	Mice	[41]
	Pharynx	Nerve fibers associated with taste buds	Mice	[41]

## Data Availability

No new data were created or analyzed in this study.

## Abbreviations

The following abbreviations are used in this manuscript:

Ascl1	Achaete-scute family basic helix-loop-helix transcription factor 1
ATP	Adenosine triphosphate
CALHM1/3	Calcium homeostasis modulator 1/3
NE	Neuroendocrine
NPJc	Nodose–petrosal–jugular ganglionic complex
NTS	Nucleus of the solitary tract
P1	Purinergic receptor type 1
P2	Purinergic receptor type 2
P2X	Purinergic receptor type 2X
P2X1	Purinergic receptor type 2X1
P2X2	Purinergic receptor type 2X2
P2X3	Purinergic receptor type 2X3
P2X2/3	Heteromeric purinergic receptor composed of P2X2 and P2X3 subunits
P2X7	Purinergic receptor type 2X7
P2Y	Purinergic receptor type 2Y
P2Y1	Purinergic receptor type 2Y1
P2Y2	Purinergic receptor type 2Y2
P2Y4	Purinergic receptor type 2Y4
P2Y6	Purinergic receptor type 2Y6
P2Y11	Purinergic receptor type 2Y11
P2Y14	Purinergic receptor type 2Y14
Pou2f3	POU class 2 homeobox factor 3
SANRA	Scale for the Assessment of Narrative Review Articles
SLN	Superior laryngeal nerve
sCPG	Swallowing central pattern generator
T2Rs	Type 2 taste receptors
TRP	Transient receptor potential
TRPA1	Transient receptor potential ankyrin 1
TRPM5	Transient receptor potential melastatin 5
TRPV1	Transient receptor potential vanilloid 1
